# Defining the Role of Solid Stress and Matrix Stiffness in Cancer Cell Proliferation and Metastasis

**DOI:** 10.3389/fonc.2018.00055

**Published:** 2018-03-12

**Authors:** Maria Kalli, Triantafyllos Stylianopoulos

**Affiliations:** ^1^Cancer Biophysics Laboratory, Department of Mechanical and Manufacturing Engineering, University of Cyprus, Nicosia, Cyprus

**Keywords:** extracellular matrix, fibroblasts, externally applied stress, growth-induced stress, *in vitro* models

## Abstract

Solid tumors are characterized by an abnormal stroma that contributes to the development of biomechanical abnormalities in the tumor microenvironment. In particular, these abnormalities include an increase in matrix stiffness and an accumulation of solid stress in the tumor interior. So far, it is not clearly defined whether matrix stiffness and solid stress are strongly related to each other or they have distinct roles in tumor progression. Moreover, while the effects of stiffness on tumor progression are extensively studied compared to the contribution of solid stress, it is important to ascertain the biological outcomes of both abnormalities in tumorigenesis and metastasis. In this review, we discuss how each of these parameters is evolved during tumor growth and how these parameters are influenced by each other. We further review the effects of matrix stiffness and solid stress on the proliferative and metastatic potential of cancer and stromal cells and summarize the *in vitro* experimental setups that have been designed to study the individual contribution of these parameters.

## Unraveling the Tumor Microenvironment

Tumor stroma and biomechanical abnormalities developed during tumor growth comprise dominant regulators of cancer progression (1–3). The tumor stroma is composed of an extracellular matrix (ECM), which consists of immune cells, fibroblasts, capillaries, and fibrillar proteins, such as collagen I, elastin, and fibronectin, as well as hyaluronan and other sulfated glycosaminoglycans (4). Fibroblasts are key regulators of ECM composition and organization and physiologically remain in the quiescent state with negligible metabolic and transcriptomic activities (5, 6). In response to tissue damage, fibroblasts become activated and are characterized by the expression of alpha-smooth muscle actin (α-SMA). In this activated state, fibroblasts overproduce ECM proteins, mainly collagen I and fibronectin, secrete cytokines and growth factors, and exert contractile forces modifying tissue architecture (5, 6).

In tumors, fibroblasts tend to acquire a constantly activated phenotype as a response to several growth factors secreted from the highly proliferative cancer cells, including transforming growth factor-β (TGFβ), epidermal growth factors (EGFs), and bone morphogenetic proteins (BMPs) (5, 6). Activated fibroblasts, which are commonly known as cancer-associated fibroblasts (CAFs), start a chronic wound healing-like response toward cancer cells, leading to an excessive accumulation of fibrillar ECM proteins, a condition known as desmoplasia (5). Under this desmoplastic reaction, CAFs continuously produce and remodel the tumor ECM increasing tumor stiffness (1, 5). Desmoplasia and ECM stiffening characterize many tumor types, especially breast and pancreatic cancers, and it usually promotes tumor progression (1, 7, 8).

As the density of cancer cells, stromal cells, and ECM constituents increase within the restricted environment of the host tissue, it leads to the development of mechanical stress (i.e., force per unit area) within the tumor (1, 3, 9–11). This stress, derived from the structural components of a tumor, is known as solid stress and can be divided into two parts. A part of it, known as growth-induced (or residual) stress, is accumulated during tumor growth by microscopic interactions among structural components of the tumor microenvironment, and it remains within the tumor even if the tumor is removed (3). These interactions might include collagen stretching by cancer cells and CAFs, and hyaluronan and cancer cell swelling to resist compression (12–15). Moreover, as tumors grow and exert forces on the adjacent host tissue, a reciprocal compressive stress is applied from the host tissue to the tumor, to resist tumor expansion (1). This stress is known as externally applied stress, and it diminishes after tumor excision (1). The total solid stress in a tumor interior is compressive (i.e., tends to reduce the size of an object), while near the interface between the tumor and normal tissue, the stress is tensile (i.e., tends to increase the size of an object) (16, 17).

## The Definition of ECM Stiffness and Solid Stress

It is not clearly defined in the pertinent literature whether matrix stiffness and solid stress refer to the same term or they are two distinct biomechanical abnormalities of a tumor that are related to each other. By definition, stiffness is a material property, which describes the extent to which a material resists deformation in response to an applied force, while solid stress is a force per unit area, which can cause either compaction (compression) or expansion (tension) of a material. In solid tumors, the stiffness is mainly determined by ECM composition and organization, while solid stress arises by the sum of the physical forces exerted during tumor growth. These forces can be generated in the subcellular level by cytoskeletal filaments that control cellular processes such as filopodia formation and extension. At the cellular level, forces are exerted by cell contractions (such as in CAFs) and cell–ECM interactions during migration of cancer and stromal cells, while at the tissue level, forces are exerted between the tumor and the host tissue (18–21).

**Figure 1:**
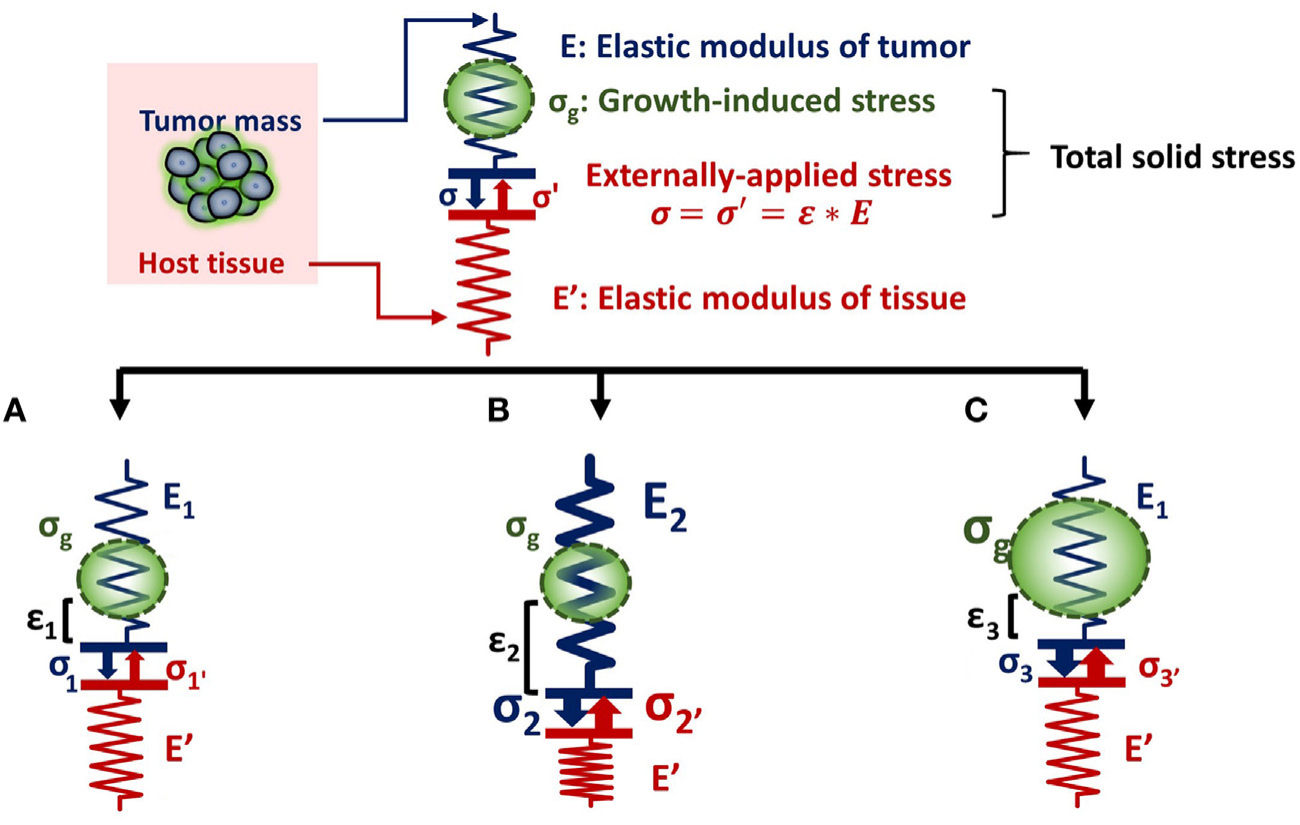
Solid stress and stiffness are two distinct biomechanical abnormalities present in the tumor microenvironment. **(A)** According to the simple analogy of a spring that obeys Hooke’s law σ = *E* ⋅ ε, when a tumor grows and pushes the surrounding host tissue of elastic modulus E’, it results in a deformation ε_1_ and a stress, σ_1_. As a consequence, the host tissue returns an equal and opposite stress σ_1_′, which is defined as externally applied solid stress (σ_1_ = σ_1_′). This externally applied stress, in combination with the growth-induced stress (σ_g_), generated from mechanical interactions within the tumor, constitutes the total solid stress transmitted in the tumor interior. **(B)** In the case that the tumor stiffens so that E_2_ is greater than E_1_ (E_2_ > E_1_), the tumor can increase in size and the deformation ε_2_ is greater than ε_1_ (ε_2_ > ε_1_). The externally applied stress (σ_2_′) and finally the total solid stress accumulated in the tumor interior are greater than that in **(A)** without any change in the growth-induced stress. **(C)** The growth-induced solid stress, however, increases during growth, while tumor stiffening might remain the same (16). In this case, the externally applied solid stress σ_3_′ can be equal to σ_1_′, but total solid stress increases. Therefore, the resultant stress transmitted in the tumor interior is greater than that in **(A)** without any change in tumor stiffness.

The relationship between tumor stiffness and solid stress can be described using the analogy of a spring of specific elastic modulus (E) that obeys Hooke’s law (Figure 1). According to the equation of Hooke’s law for linear elastic materials, σ = *E* ⋅ ε, when a tumor of elastic modulus E grows and pushes the surrounding host tissue of elastic modulus E′, it causes a deformation ε_1_ and a subsequent stress σ_1_. As a consequence, the host tissue returns an equal and opposite stress σ_1_′, the so-called externally applied solid stress. At the same time, growth-induced solid stress is accumulated in the tumor interior owing to interactions among tumor components (Figure 1A). Thus, the total solid stress accumulated intratumorally is the sum of the externally applied and the growth-induced solid stress. In the case that the stiffness of the tumor E_2_ is greater than E_1_, then the tumor can displace the host tissue with a greater deformation and the externally applied solid stress σ_2_ can be greater than σ_1_ (Figure 1B). Therefore, in this case, a solid tumor creates a stiffer matrix to push against the normal tissue and grow in size. Indeed, it has been demonstrated using mathematical modeling that the stiffness of a solid tumor should be at least 1.5 times greater than that of the host tissue, in order for the tumor to displace the tissue and grow (14).

As for the growth-induced solid stress, it increases during tumor growth, while the matrix stiffness might stop changing (16, 17). In this case, the further increase in total solid stress accumulated intratumorally can become less depended on matrix stiffness (Figure 1C). This hypothesis was confirmed by an elegant study by Nia et al. (16), showing that the total solid stress transmitted into the cells can depend only in part on tumor stiffness, and thus, the two terms should not be used without a distinction. Specifically, Nia et al. found that primary pancreatic tumors exhibited larger stresses compared to those in metastatic sites, while the opposite effect was observed for colon tumors (16). Interestingly, tumor stiffness was similar in the primary and metastatic tumor for both the pancreatic and colon cancer models, showing that tumor stiffness and solid stress are not necessarily coupled to each other. In addition, they found that solid stress increased in breast tumors of larger size despite the fact that stiffness did not change with tumor size. In line with our analysis, these observations can be explained by the fact that growth-induced solid stress generated owing to microscopic interactions among structural components in the tumor interior contributes to the accumulation of an additional to the externally applied solid stress. Therefore, the effects of matrix stiffness and solid stress on tumorigenesis and metastasis should be studied separately (22). Following, we provide a summary of these effects on cancer and stromal cell behavior, elaborating on the less studied contribution of solid stress and the pertinent experimental setups.

## Effects of Matrix Stiffness on Cancer and Stromal Cells

**Figure 2:**
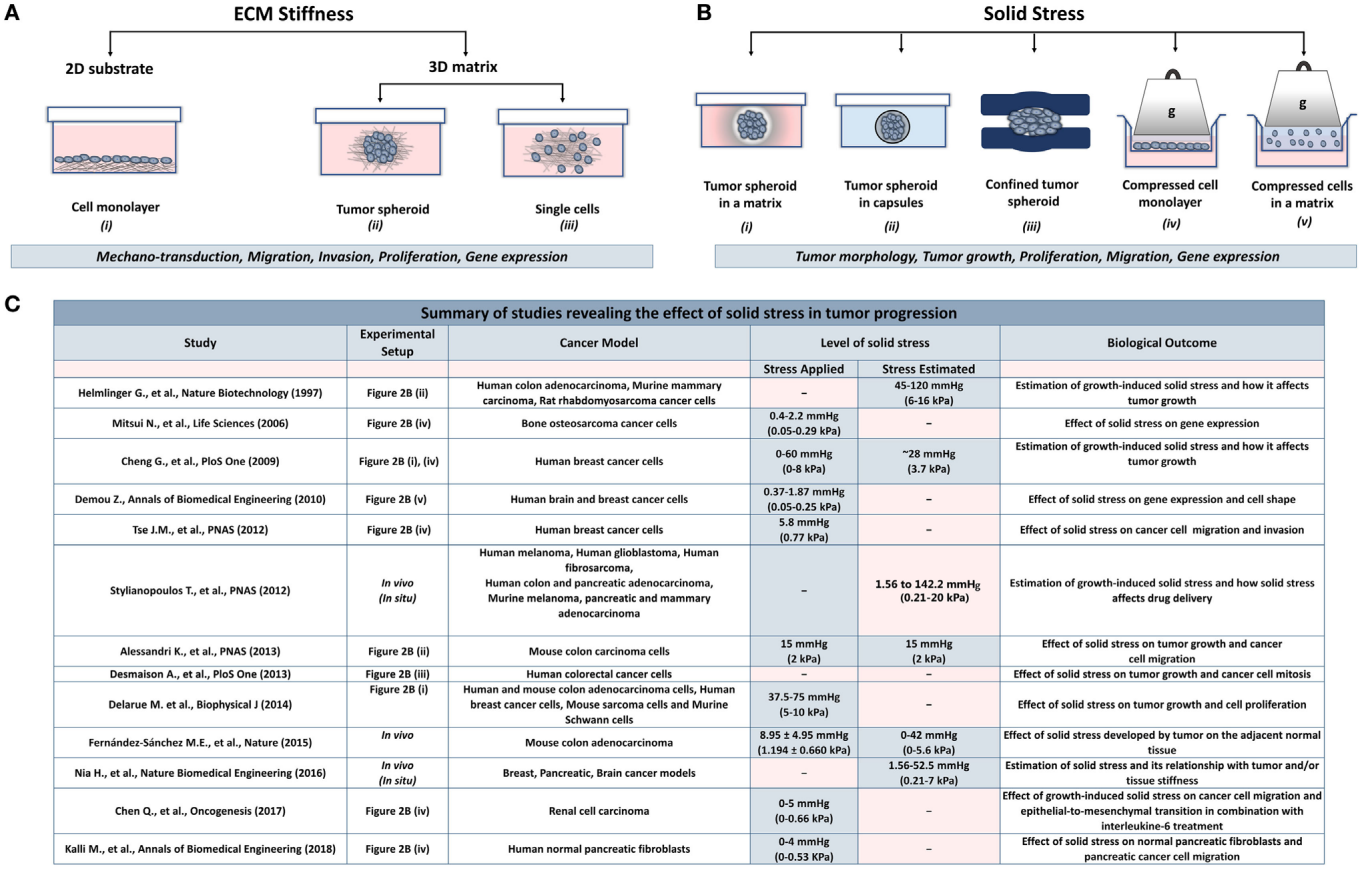
Experimental methods employed to analyze the effects of stiffness and solid stress on cancer and stromal cells *in vitro*. **(A)** Experimental setups studying the effect of ECM stiffness on cancer and stromal cells. There are two-dimensional models (2D) consisting of (i) a cell monolayer seeded on coating substrates (e.g., collagen type I or fibronectin) and three-dimensional models (3D) consisting of (ii) tumor spheroids or (iii) single cells embedded in a matrix (e.g., collagen type I, matrigel). Both models were aimed to investigate the effect of changes in extracellular rigidity on the transduction of mechanical signals into the cells as well as on the migration, invasion, proliferation and gene expression of cancer and stromal cells **(B)** Experimental setups studying the effect of solid stress on cancer and stromal cells. Setups include tumor spheroids that grow within (i) a polymer matrix, (ii) within elastic capsules, or (iii) in a confined polymer device. (iv,v) The setups are composed of cells seeded on the inner chamber of a transwell insert on the top of which an agarose cushion is placed or are embedded in a polymer matrix. A piston with adjustable weight applies a predefined and measurable compressive solid stress on the cells. These models provided useful information about the direct effect of solid stress on tumor growth and morphology as well as on cancer cell proliferation, migration, and gene expression. **(C)** A summary of *in vitro* and *in vivo* studies for the effect of solid stress in tumor progression.

The effect of ECM stiffness on cancer and stromal cells has been studied using *in vitro* two-dimensional substrates (2D) and three-dimensional tumor analogs (3D). In 2D models, cells are seeded on coating substrates such as collagen or fibronectin (23–26), while the 3D models include single cells or tumor spheroids embedded in gels composed of collagen or matrigel (27–34) (Figure 2A). In both cases, stiffness is increased by changing the protein density or the degree of crosslinking of the matrix to study the effects of ECM-originating mechanical cues on cancer and stromal cells.

Matrix stiffness can activate intracellular signaling pathways to regulate cellular behavior. Cancer cells recognize the increase in ECM stiffness and respond by generating increased traction forces on their surroundings through actomyosin and cytoskeleton contractility (9, 35, 36). Moreover, the changes in matrix rigidity are sensed and transmitted intracellularly through mechanosensors such as p130 CRK-associated proteins, growth factor receptors, or integrin-ECM adhesion plaques (9, 10, 23, 35, 37–40). These mechanosensors can recruit focal adhesion molecules such as FAK, SRC, paxillin, RAC, RHO/RAS GTPases, and Rho-associated kinase to trigger signaling cascades and cytoskeleton organization (9, 10, 35, 36, 39, 41–44). These cascades finally regulate gene expression and induce quantifiable changes in cell shape, survival, migration, and invasion (9, 35, 39, 42). For example, it has been shown that tissue stiffness activates the nuclear translocation of the transcription factor TWIST1 in breast cancer cells, which inhibits the expression of E-cadherin and promotes cell invasion (35, 45). Furthermore, in a 3D model consisting of breast tumor spheroids growing in collagen matrix, the Ras suppressor-1 (RSU-1), a cell-ECM adhesion protein, was shown to be upregulated as a response to increasing stiffness. Interestingly, tumor spheroids knockdown for RSU-1 or actin polymerization regulator (VASP) lost their invasiveness through the 3D matrix (46, 47). Matrix stiffening is also shown to induce fibroblast activation and migration, which leads to a fibrotic response setting a positive feedback to matrix stiffness (13, 15, 35, 48, 49). However, in these studies, it cannot be distinguished explicitly whether the observed effects are emerged by increased cell-ECM adhesion sites owing to increased ECM density or by stiffness-induced solid stress generation.

## Effects of Solid Stress on Cancer and Stromal Cells

While the role of ECM stiffness in cancer and stromal cells is actively studied, data regarding the effect of solid stress on tumor progression are elusive. There are several experimental setups mimicking the solid stress developed in the tumor microenvironment. These setups include models consisting of tumor spheroids growing in a confined environment that causes the development of solid stress (50–57) and models employing a transmembrane pressure device that applies a mechanical compression on a cell monolayer or on single cells embedded in a matrix (51, 58–60) (Figure 2B).

Regarding the first method, cancer cells are grown as spheroids in a polymer gel (e.g., agarose), which leads to the development of solid stress that resists to spheroid expansion (Figure 2B, i). Helmlinger et al. (55) using spheroids of colon adenocarcinoma cells estimated that the accumulated solid stress was in the range of 45–120 mmHg (6–16 kPa), depending on the concentration of the agarose gel and the size of the spheroid. In an analogous study, Cheng et al. (51) estimated the solid stress to be ~28 mmHg (~3.73 kPa) when mammary carcinoma cell spheroids were growing in a 0.5% agarose matrix. Recent *in vivo* measurements of breast, colon, pancreatic, and brain tumors estimated that the growth-induced stress is in the range of 1.56–142.4 mmHg (0.21–20 kPa) (3, 11, 16, 54). Differences in the magnitude of solid stress among *in vitro* studies and between *in vitro* and *in vivo* methods should depend on the tumor model and the experimental procedure used in each study. However, the conclusion that increasing compressive stress inhibits tumor growth is common (51, 52, 55, 57), while this effect was reversed when loads were removed (51, 55). It was also observed that solid stress can regulate tumor morphology since mechanical loads can induce apoptotic cell death in regions with high compressive stress and allow proliferation in low-stress regions of the tumor spheroid, suggesting that anisotropic loads result in anisotropic tumor growth (51).

More recent studies developed novel techniques to mimic solid stress during tumor growth in the absence of a matrix. Alessandri et al. (50) employed a microfluidic method based on the encapsulation and growth of cells inside permeable, elastic, and hollow microspheres (Figure 2B, ii). This approach offered the ability to produce size-controlled multicellular spheroids growing in confined conditions. They found that the confined spheroids exhibited a necrotic core compared with the unconfined spheroids. In contrast, peripheral cells were more proliferative and migratory, suggesting that mechanical cues from the surrounding microenvironment may trigger cell invasion from a growing tumor (50). Desmaison et al. (53) designed polymer polydimethylsiloxane microdevices to restrict the growth of spheroids and subsequently to induce the development of mechanical stress (Figure 2B, iii). They showed that the mitosis of mechanically confined spheroids was suppressed compared to spheroids grown in suspension (53). Furthermore, it was demonstrated that a population of cells within the confined tumor spheroids was arrested at mitosis, which was due to the inhibition of bipolar spindle assembly (53). Later, Fernández-Sánchez et al. (54) developed a method that allows the delivery of a defined mechanical pressure *in vivo* by subcutaneously inserting a magnet close to the mouse colon. The implanted magnet generates a magnetic force on ultramagnetic liposomes stabilized in the mesenchymal cells of the connective tissue surrounding colonic crypts after intravenous injection (54). The induced pressure was similar in magnitude to the endogenous stress (54), in the order of 9.0 mmHg (1.2 kPa), without affecting tissue stiffness, as monitored by ultrasound strain imaging and shear wave elastography (54). The magnetic pressure stimulated Ret activation and the subsequent β-catenin phosphorylation, impairing its interaction with E-cadherin in adherens junctions (54). These data suggested that tumor progression could be driven by signaling pathways that are directly activated by mechanical pressure.

To study the effect of a predefined solid stress on cancer cells, the transmembrane pressure device has been introduced (Figure 2B, iv). Setups employed consist of a transwell insert that fits in a well of a 6-well culture plate. The insert is separated in the lower chamber containing culture medium and the upper chamber containing the cell monolayer. A piston of a preferable weight is applied on the cell monolayer, while water, nutrients, and oxygen from the culture media are diffused through the pores of the transmembrane. This device provides a tool to mimic solid stress in a predefined manner according to the stress magnitudes measured in native tumor tissues.

Cheng et al. (51) used this device to study the effect of solid stress on murine mammary carcinoma cells. In this study, they applied a stress ranging from 0 to 60 mmHg (0–8 kPa), and they observed increased apoptosis with increased stress levels. In a following study, they used the same experimental setup to study the migration of cancer cells using a scratch wound assay (60). They applied a stress of 5.8 mmHg (0.77 kPa), and concluded that in these levels of compression, cancer cells stopped proliferating and started to create a leader cell formation, which allowed them to move toward the scratch having an invasive phenotype. Mitsui et al. (59) used a similar device for bone osteosarcoma cells to identify the effect of compressive stress on the expression of matrix metalloproteinases and plasminogen activators. They observed enhanced protein and mRNA levels of these molecules under low mechanical compression of bone cells (0–2.20 mmHg/0–0.29 kPa) (59). Recently, Chen et al. (61) observed increased migration and mesenchymal-like phenotype of renal carcinoma cells that were compressed by 0–5.0 mmHg (0–0.66 kPa), while Kalli et al. (62) found that normal fibroblasts become activated as a response to solid stress to promote pancreatic cancer cell migration.

Another device that was developed to study the effect of solid stress in a more realistic way involved the use of single cancer cells growing in an agarose matrix (Figure 2B, v). This device was composed of two custom-made parts, the well pressor and the optic pressor (58). Both devices consisted of a chamber containing a 3D gel with single cells embedded, a screw and a nut for pressure application, and their housing support. Specifically, the well pressor applied a strain that compressed the cell-contained agarose gels to 50% of their original volume. This stress was estimated to be ~0.37 mmHg (~0.05 kPa), much smaller than loads measured by other studies (3, 51, 55, 58). However, this stress was sufficient to cause differential expression profiles of metastasis-associated genes in glioblastoma and breast cancer cells. In addition, the optic pressor provided quantifiable changes in cell circularity and orientation with respect to the direction of the applied force (58).

Collectively, these *in vitro* studies suggest that mechanical forces can regulate tumor morphology, tumor growth, and metastatic potential of cancer cells in the absence of matrix stiffness. However, as indicated in Figure 2C, there is a discrepancy among the levels of solid stress applied or estimated in the pertinent studies due to the variability of the experimental procedures and the cancer models used. Therefore, it should be given special attention when performing experiments to study the effect of solid stress on tumor progression, taking into account the estimations derived from *in vivo* studies.

## Conclusion and Future Perspectives

In light of recent studies showing that increased matrix stiffness and elevated solid stress are two distinct tumor abnormalities, and given the fact that most pertinent studies are focused on the effects of stiffness, it becomes clear that scientific efforts need to focus on the implications of solid stress in tumor progression and metastasis (16, 22).

Regarding the implications of tumor stiffness in tumor progression, most pertinent *in vitro* models include only cancer cells and ECM matrix. However, tumor stiffness might also depend on the presence of stromal CAFs that continuously interact with the fibrillar proteins. CAFs-ECM interactions remodel the ECM organization and fibers orientation for cancer cells to migrate and invade into the matrix (1, 63, 64). Regarding the effects of solid stress on tumor progression, further studies are required to shed light upon the mechanisms by which solid stress is transmitted and guides cellular behavior of both cancer cells and CAFs. Moreover, CAFs exert contractile forces that contribute to the accumulation of solid stress in the tumor interior. Therefore, it is necessary to include both cell types when solid stress and ECM stiffness are being studied.

It has been also shown that CAFs dynamically interact with cancer cells to promote tumor progression (62, 64). In fact, CAFs mediate the invasiveness of colon, pancreatic, and breast cancer cells when co-injected into mice (64–68), while breast and prostate tumors containing CAFs grew faster than tumors injected with normal fibroblasts (69, 70). Nevertheless, there is no pertinent study taking into account the effect of ECM stiffness and solid stress on the interaction of cancer cells and CAFs and *vice versa* the implication of tumor-stromal interactions in ECM stiffening and solid stress accumulation.

Concerning the complexity of the tumor microenvironment, new experimental setups consisting of cancer cells, CAFs, and changes in matrix stiffness and solid stress, in combination or separately, should be introduced to broaden our knowledge about the role of each component in the evolution and malignancy of cancer.

## Author Contributions

MK and TS planned and wrote the manuscript of this mini review.

## Conflict of Interest Statement

The authors declare that the research was conducted in the absence of any potential conflict of interest.
